# Mechanically guided cell fate determination in early development

**DOI:** 10.1007/s00018-024-05272-6

**Published:** 2024-05-30

**Authors:** Delan N. Alasaadi, Roberto Mayor

**Affiliations:** Department of Cell and Developmental Biology, University College London, Gower Street, London, WC1E 6BT UK

**Keywords:** Cell fate, Mechanobiology, Embryogenesis, Cell signaling

## Abstract

Cell fate determination, a vital process in early development and adulthood, has been the focal point of intensive investigation over the past decades. Its importance lies in its critical role in shaping various and diverse cell types during embryonic development and beyond. Exploration of cell fate determination started with molecular and genetic investigations unveiling central signaling pathways and molecular regulatory networks. The molecular studies into cell fate determination yielded an overwhelming amount of information invoking the notion of the complexity of cell fate determination. However, recent advances in the framework of biomechanics have introduced a paradigm shift in our understanding of this intricate process. The physical forces and biochemical interplay, known as mechanotransduction, have been identified as a pivotal drive influencing cell fate decisions. Certainly, the integration of biomechanics into the process of cell fate pushed our understanding of the developmental process and potentially holds promise for therapeutic applications. This integration was achieved by identifying physical forces like hydrostatic pressure, fluid dynamics, tissue stiffness, and topography, among others, and examining their interplay with biochemical signals. This review focuses on recent advances investigating the relationship between physical cues and biochemical signals that control cell fate determination during early embryonic development.

## Introduction

 Embryologist William Brooks stated, “The greatest of all wonders of the material universe: the existence, in a simple, unorganized egg, of a power to produce a definite adult animal” [1]. This statement poses a fundamental question: What laws dictate an “unorganized egg” to become specialized and highly organized cells that elicit tissue-specific function through a process known as cell fate determination? Explicitly how an unorganized multicellular embryo gives rise to, for example, red blood cells that distribute oxygen to billions of cells and cells that specialize to become neurons, the building block of the nervous system [1]. Since William Brooks’s reflection in 1883, scientists unveiled much of the morphogens and molecular programs that influence cell fate determination [2–4]. Indeed, recent advancements in molecular biology allowed further in-depth examination of the molecular aspects in ever-increasing detail [5–7]. These methodologies produced an enormous amount of data that, taken together, lead to the notion that the cell fate determination process is a complex and challenging task. However, in parallel to the biochemical and molecular approaches, researchers have investigated the role of physical laws that derive morphogenesis as a possibility to untangle the intricate interplay of biochemical and molecular regulation of cell fate determination [8, 9]. Physical forces regulate all stages of development, from fertilization, morphogenesis, body plan formation, and organogenesis [8, 10]. Advancements in biophysical approaches have demonstrated that mechanical forces are not restricted to cell motility and behavior but also to fate determination [10]. This review discusses the approaches to cell fate determination and understanding how mechanical forces interplay within biological systems during cell fate in early embryogenesis.

## Road map to biomechanics

Despite understanding how genes and biochemical signaling direct cell fate, which has revolutionized the study of cell biology and embryogenesis over the last century, new insights into how mechanical cues regulate cell fate have gained considerable attention over the last two decades [11]. Indeed, during embryogenesis, extrinsic mechanical inputs such as fluid flow, sheer stress, hydrostatic pressure, tension, compressive forces, and others, as well as intrinsic forces such as cell density, shape, and extracellular elasticity and topography, are essential for cell fate, motility, and behavior [10, 12].

Therefore, a model that outlines how physical laws dictate biological processes is crucially required. Richard Feynman adds in The Character of Physical Law, *“You can recognize truth by its beauty and simplicity… inexperienced students, make guesses that are very complicated, and it sort of looks as if it is alright, but I know it is not true because the truth always turns out to be simpler than you thought”* [13]. Implementing Richard Feynman’s perspective on the role of mechanobiology on cell fate, we can see this complex, interwind, and detailed field into three axes: *Active input* (the strains and stresses exerted on cells), *Passive inputs* (the material properties of cells and the surrounding environment), and the bio-mechano-chemical *cellular response* mediated by the impingement of mechanical stimuli [14].

**Fig. 1:**
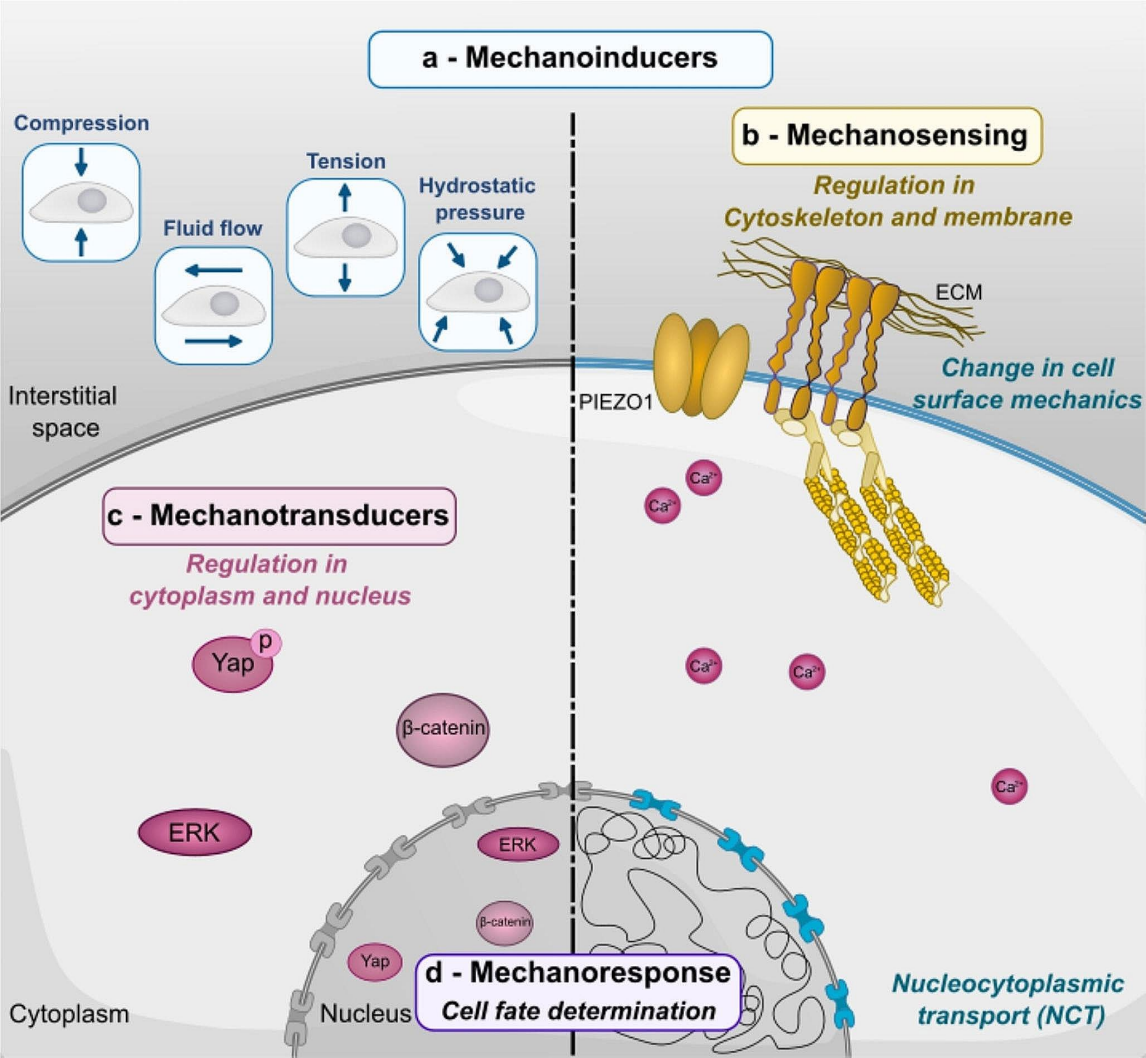
Components of mechanotransduction pathway in cell fate determination. **a**, Mechano-inducer elements (in blue) that apply physical force. The physical source can be extrinsic (e.g., compressive force, fluidic pressure, among others) or intrinsic (e.g., change in cell membrane property/tension, nuclear to cytoplasm shuttling, among others). **b**, Mechano-sensing elements (in yellow) that respond to the physical force applied upstream (often) and initiate a cascade of biochemical reactions downstream. **c**, Mechano-transducer elements (in pink) are proteins or ions that are part of the signaling pathway and have the ability to respond to mechano-sensing elements. **d**, Mechano-response, a cellular response towards the activation of the mechanotransduction pathway that leads to change at the transcriptional level, ultimately, in this case, to fate regulation

The mechanical landscape is intricate; both extrinsic and intrinsic cues often cannot be decoupled (Fig. 1). Cells perceive mechanical signals via mechanosensitive elements on the cell surface, such as membrane channels (piezo1/2) [15, 16], integrins, focal adhesions [17], intracellular components; cytoplasmic proteins [18]; in addition, the nucleus has been recognized as an important mechanosensor, which we will describe later [19]. Mechanical activation at the surface promotes the cytoskeleton to respond to counterbalance the force by increasing or decreasing contractility (Fig. 1). Tension in the cytoskeleton controls mechanotransducers (Fig. 1c) that mediates downstream transcriptional activity that controls cellular response, such as cell fate [20, 21]. However, this mechanotransduction model is not unidirectional as a direct interaction between the nucleus and cytoskeleton plays a role in how cells perceive these mechanical cues, leading to a feedback loop on transcriptional activity [20, 22]. This current mechanotransduction pathway is not the full picture as it places the cytoskeleton as a central component to translate physical forces into biological outcomes. However, this might not be the case, as recent evidence shows the ability of the nucleus (Fig. 1d) to mediate the mechanotransduction pathway independently and regulate cellular responses toward physical forces [19]. Thus, a revamped model encompassing mechanical stimuli into a biological outcome might be crucial to move forward.

The earliest biophysical experiments *in vitro* investigating the potential role of physical force on cell fate were conducted on mesenchymal stem cell (MSC) differentiation. Adipogenic or neuronal differentiation from MSC is promoted on soft matrices (mimicking brain stiffness), whereas stiff matrices (mimicking bone stiffness) induce osteoblast or myocytic differentiation [23–25]. These findings provided a paradigm shift, now considered a cornerstone example, in our understanding of the role of physical force on cell fate. Moreover, if MSCs are cultured in neurogenic stiffness (0.1-1 kPa) and after three weeks are incubated with myogenic or osteogenic media, inductive signals are overridden, and neurogenic fate is maintained by MSC [26]. These studies suggest that intrinsic mechanical input (ECM stiffness) is sufficient to drive MSC fate; nevertheless, induction media can enhance this response. Furthermore, these studies strengthen the notion of mechanical input interplay with biochemical signals to prompt cellular fate [24, 26, 27]. With these validations of the role of physical forces on cell fate, the question at hand has shifted from *whether mechanical cue has a role in cell fate determination* to *how cells sense and incorporate physical forces into a differential biological state*. To address this idea, researchers found that on stiff material, both spreading and actomyosin contractility of MSC leads to increased WNT and ERK signaling that promotes osteogenic fate [28]. These findings were validated by controlling the spreading of MSC and showing that it is sufficient to control the fate of these cells [29]. This outcome is pivotal as it infers that the geometric shape of cells directly controls their fate. Moreover, by investigating the molecular bases of this outcome, scientists demonstrated that lineage choices depend on traction force applied to integrin-associated complexes (α_v_ and α_5_) (Fig. 1a, b) [30]. Furthermore, the geometry of tissues plays a vital role in epithelial fate [31]. Researchers engineered, via pressurization, a curved epithelial monolayer with different shapes (e.g., rectangle, circler, or ellipsoidal) and showed that different strains (stress) are applied on these epithelial monolayers in a geometry-dependent manner, leading to cell realignment and ultimately change in epithelial fate [31]. Collectively, these findings implicate the role of mechanical cues on cell fate, and these forces regulate cells at the transcriptional level dependent on the adhesion complex. Further investigations showed that MSC fate depends on the extracellular matrix independently of stiffness [32]. Indeed, MSC differentiates into adipocytes in a non-degradable 3D matrix, whereas it differentiates into osteogenic in a degradable 3D matrix [33, 34]. This biomechanical developmental approach highlights the functional ability of MSCs to remodel their environment to promote osteogenic fate [33]. The biomechanical role of cell fate *in vitro* is not restricted to MSCs. The differentiation of mouse pluripotent stem cells (PSCs) into endoderm depends on traction forces. Reducing traction forces led to decreased transforming growth factor-β (TGFβ) signaling required for endoderm fate determination. This change in physical force, ultimately regulating a signaling cascade, is biomechanically sensed by integrins such as α5β1 and α3β1 [35]. Cell fate is not restricted to traction forces, as physical force, nor mediated by TGFβ only [36, 37]. Certainly, further evidence showed that, for example, fibroblast growth factor (FGF), WNT, and Notch are mechanosensitive pathways to several physical cues, such as stiffness, sheer flow, and tension, among others [36, 37]. For instance, fluid sheer stress in mouse embryonic stem cells relocalizes β-catenin from the adherence junction to the nucleus to regulate stemness [38]. Researchers utilizing a human pluripotent stem cell micropatterning system demonstrated that direct mechanical force on tissue (e.g., stretching) increases cell-adhesion tension, leading to relocalization of β-catenin and activation of Wnt signaling, subsequently regulating mesoderm specification [39] and that patterning of neuroepithelial/neural plate border in a BMP-SMAD dependent manner is also controlled by physical forces (increase in stretching) [40]. Moreover, fluid sheer stress promotes MSC differentiation into osteogenic fate dependent on Ca^2+^ and MAPK/ERK signaling [41, 42]. Additional evidence of how mechanical cues control stem cell fate has been recently reviewed elsewhere [27].

Further examination of the molecular components and functions as mechanosensors revealed yes-associated protein (Yap) (Fig. 1c) and, more recently, Piezo1 membrane ion channel (Fig. 1b) as strong candidates to promote cell fate determination. For example, Yap-depleted MSC cultured on a stiff substrate inhibited osteogenic and enhanced adipogenic fate [43, 44]. In addition, Piezo1 activation is mediated by the stiffening of the brain due to aging, promoting the influx of Ca^2+^ and subsequently initiating mechanosensitive pathways that control the proliferation and differentiation of oligodendrocyte progenitor cells (OPC) [45, 46]. Although these studies point out the molecular components within the signaling cascade that regulate the mechanotransduction pathway, a detailed mechanism on how precisely these elements control gene regulation that determines cell fate is missing. In addition, these findings raise the question of whether mechanosensitive pathways could regulate other biochemical signals. In the next section, we examine recent evidence of the interplay of forces with chemical cues in early development to regulate cell fate.

### The interplay between chemical and mechanical cues on cell fate determination

Mechanotransduction is a biological phenomenon in which cells sense physical force(s) and elicit a biochemical response (e.g., alteration in phosphorylation state and/or protein translocation or confirmational change) and, in some cases, leading to gene regulation altering biological outcome (cellular phenotype, migration, and/or behavior) [15, 47]. The mechanotransduction process has been investigated theoretically and experimentally in the past decades, leading to a more precise definition and identification of some mechanotransduction components - known as mechanosensors. Certainly, mechanotransduction can be defined as the single-point convergence and translation of physical force into biochemical responses [47]. In addition, researchers identified the source and the type of physical cues generated within biological systems. Indeed, examinations in embryogenesis have found that several developmental processes can contribute to extrinsic or intrinsic mechanical cues, such as tissue growth, movement, and rearrangement, among others. These mechanical inputs can be regulated by cell-cell contact as seen in tissue compaction in mice [48, 49], fluid-to-jamming transition as described for body axis elongation [50, 51], or by increasing cell density of the head mesoderm, which is sensed by neural crest cells initiating migration [52]. Hence, here we examine the single-point convergence of various physical stimuli that initiate the mechanotransduction pathway regulating cell fate during development.

**Fig. 2:**
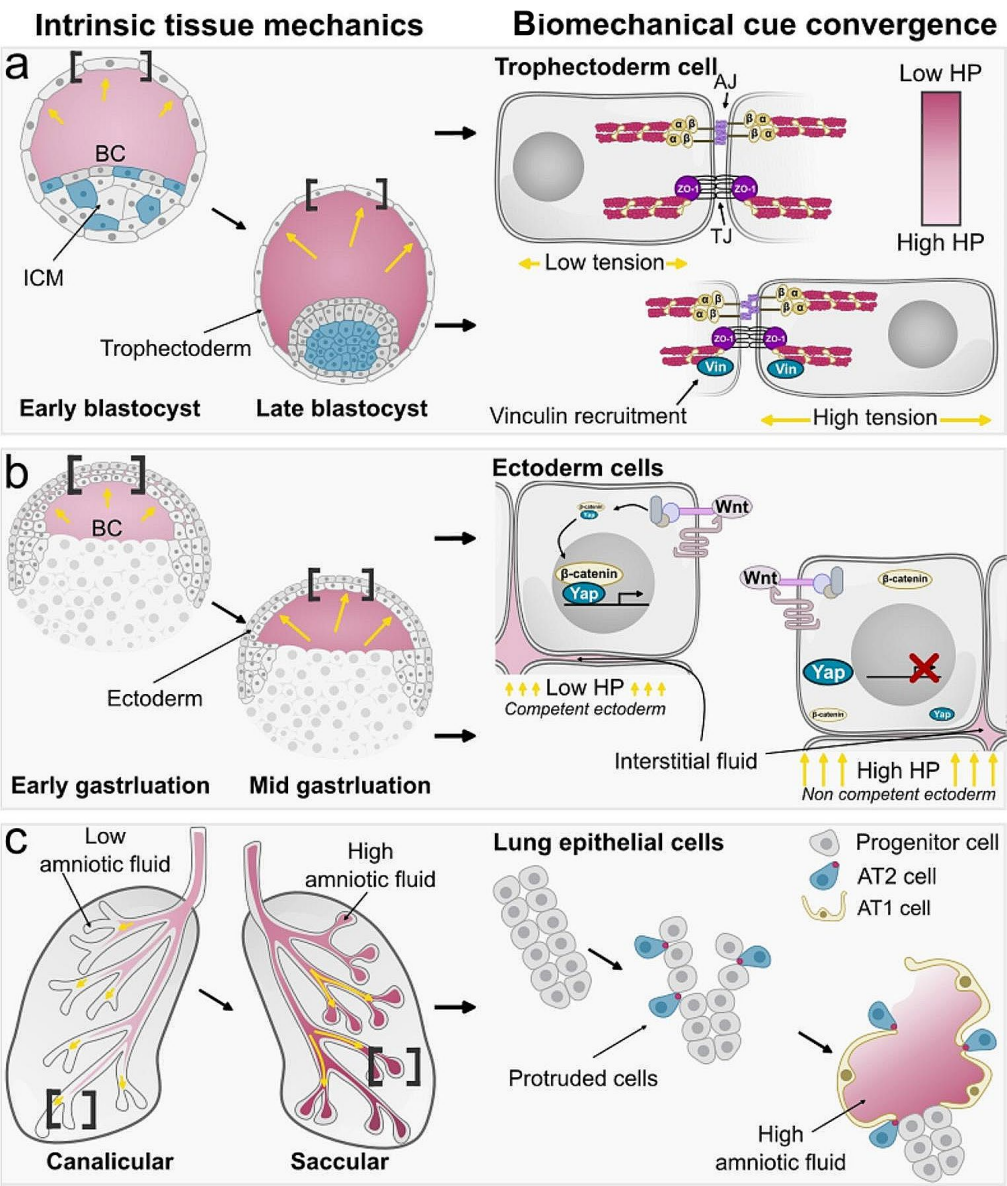
Mechnotransduction of physical force into biochemical signals. **a**, An upsurge of tension in mice trophectoderm is mediated by the increased hydrostatic pressure of the blastocoel cavity (BC) that leads to vinculin recruitment to tight junctions. **b**, Increase in *Xenopus* embryos blastocoel volume and hydrostatic pressure controls the ectoderm competence to respond to Wnt signaling mediated by mechanosensor Yap. **c**, Embryonic alveolar epithelial cell differentiation, controlled by both cellular protrusion and mechanical cues developing from amniotic fluid inhalation. At the distal airway tips, prior to the arrival of inhaled amniotic fluid, alveolar progenitor cells begin protruding, leading to reduced apical surface area and the accumulation of apical myosin (red). Nonprotruding cells are flattened and differentiate into AT1 cells by mechanical cues; in contrast, protruding cells maintain their cuboidal shape and differentiate into AT2 cells

The human body mostly consists of water, while 75% is located within cells. Approximately 75% of the remaining 25% is located extracellularly and interstitially, and this interstitial fluid during embryogenesis is in a continuous turnover, expansion, or reduction [53]. This extracellular fluid can be a source of physical force (hydrostatic pressure), which has gained researchers’ interest for its ability to remodel tissue and its possible role in fate determination [54]. Remodeling of epiblast and primitive endoderm is linked to lumen morphogenesis in murine embryos between day E3.5 and E4 [55]. Mechanistically, the continuous build-up in fluid pressure led to fractures in cell-cell contact generating microlumens in mice embryos, followed by directed contractility, mediating the formation of the embryonic cavity called blastocoel [55]. Arguably, the morphodynamic movements of tissues could be a source of mechanical cues that regulate mice’s early embryonic fate. Definitely, lumen volume controls the fate of epiblast primitive endoderm [56] and trophectoderm [57]. The formation and the expansion of the blastocoel cavity in the murine embryo correlates with the secretion of FGF4, required for epiblast primitive endoderm fate specification, and any pharmaceutical or mechanical perturbation of the formation of this cavity leads to impairment in the specification of these embryonic tissues [56]. In the case of trophectoderm, scientists found that a two-fold increase in lumen pressure increases cortical tension and stiffness of this layer, leading to vinculin mechanosensing (via ECM) and remodeling of tight junction during cell division, which in turn controls cell positioning and the fate of trophectoderm (Fig. 2a) [57]. The detailed mechanosensing mechanism by which the lumen controls cell specification remains poorly understood. However, one possibility is that altering lumen volume changes the availability of soluble factors required for cell fate determination [56, 57]. Later, an investigation in *Xenopus* embryos that examines the impact of hydrostatic pressure on embryonic competence (the ability to respond to inductive signals) uncovers that a decrease in competence for neural crest induction coincides with an elevation of pressure inside the blastocoel, a cavity adjacent and in contact to the prospective neural crest [58]. Indeed, Alasaadi and colleagues, through *in vivo* hydrostatic pressure manipulation, demonstrate that increased pressure increases the cell packing (crowding) of ectodermal cells which inhibits Yap signaling and impairs Wnt activation, a signal essential for neural crest induction [58]. Authors further showed the effect of changing hydrostatic pressure on neural crest induction extends to mouse embryos and human cells in addition to *Xenopus*, suggesting a conserved mechanism across vertebrates whereby convergence of tissue mechanics and inductive signaling pathway control embryonic competence [58] (Fig. 2b). Further evidence of how intrinsic tissue mechanics converge with signaling pathway derives organogenesis is shown when a localized increase in cell proliferation creates spatial compressive forces (circular patterns of mechanical anisotropy) which control Yap’s activity, ultimately orchestrating the development of the enamel knot [59]. Additionally, it was found that in the early and late mice, lung organogenesis, a transmural fluid-mediated pressure, influences cell differentiation. Isolated early embryonic lungs and cultured *ex vivo* exhibited branching and development that resembles embryonic development under high transmural pressure [60]. In contrast, abnormal lung development and branching are noted under low transmural pressure [60]. In addition, fetal breathing translocates fluids toward the branching tips of the lung [61]. Alveolar progenitor that resides in the branching tips constricts their apical surfaces, protecting them from this pressure and giving rise to alveolar type ll cells; in contrast, adjacent cells that are exposed to hydrostatic pressure give rise to thin and elongated alveolar type l cells (Fig. 2c) [61]. The different fate outcomes synergistically depend on sensing the pressure and growth factors (FGF10/FGFR2) [61]. Mechanistically, FGF10 initiates the ERK pathway, forming protrusions that protect alveolar progenitors from pressure. Thus, the fate of these progenitors depends on their ability to respond or not to mechanical cues in a biochemical-dependent manner (Fig. 2c) [61]. In addition, it was demonstrated that periodic deflation and inflation of *Hydra* tissue is vital for regenerating the head organizer and patterning the epithelial lumen [62]. Ferenc and colleagues found that mechanical inflation and deflation cycles lead to tissue stretching that correlates with the secretion of the Wnt3 ligand, which feeds positively into the activation of WNT signaling that specifies patterning of the *Hydra* body axis and oral pole [62]. Collectively, these studies shed light on the importance of fluid-driven biomechanics in cell fate determination during embryogenesis. However, a detailed examination of the mechanotransduction pathway that translates hydrostatic pressure into biochemical signals is required. Certainly, a recent study in *Hydra* proposed that the canonical WNT pathway spatiotemporally promotes extracellular matrix (ECM) remodeling during axis patterning [63]. This provokes the notion that head organizer cells sense deflation and inflation via ECM (stiffness-dependent mechanotransduction pathway).

**Fig. 3:**
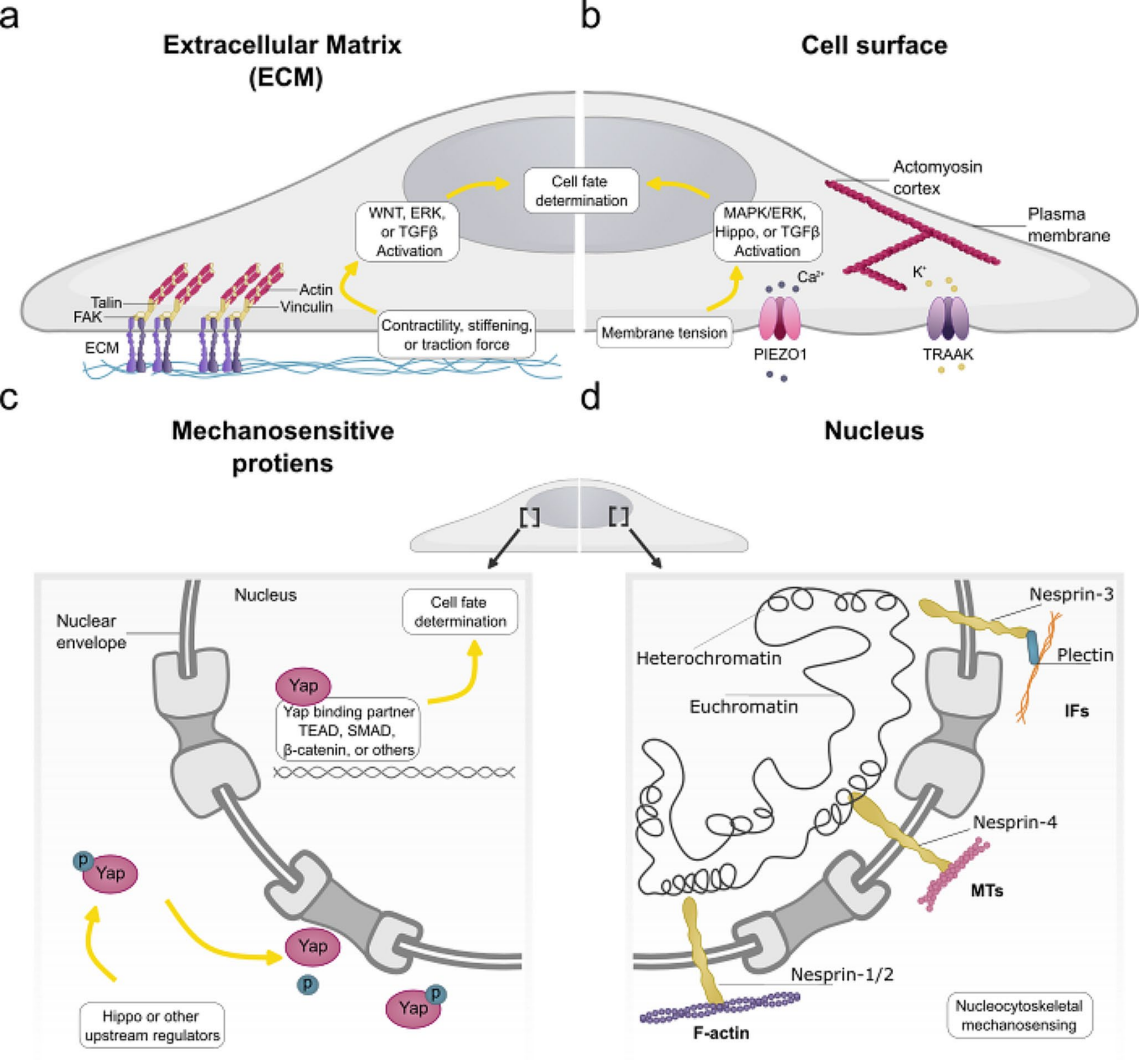
Convergence of physical cues into biochemical pathways. **a**, Extracellular Matrix (ECM) promotes activation of signaling pathways (WNT, ERK, and others) that regulate cell fate via mechanosensory such as vinculin. **b**, Membrane deformation (a change in cell surface mechanical property) leads to activation of mechanosensitive ion channels such as Piezo1 and controlling downstream effectors. **c**, Transducers of mechanical cues, such as Yap, translocated to the nucleus regulating transcriptional activity. **d**, Nucleus can act as a mechanotransducer of force by mediating protein translocation and as a mechanoinductive where it remodels cellular shape or elasticity to elicit a biological response

The Extracellular Matrix (ECM) influences various cellular behaviors (fate determination, proliferation, migration, and cell shape) by providing physical and chemical signals [43, 64–69]. From a biophysics perspective, the ECM contributes to the elasticity properties of tissue, the physical principle that provides the framework for how the ECM controls various biological behaviors. Elasticity of tissue is defined as its ability to resist an extrinsic physical force that leads to material deformation, a property measured/expressed by *Stiffness* [43, 64, 66–68]. The stiffness of various cell lines and biological systems has been implicated in different biological outcomes (Fig. 3a). Indeed, the mechanotransduction pathway of how different stiffness, both *in vivo* and *in vitro*, has been thoroughly examined [43, 65, 70]. Recent evidence demonstrates that embryonic ECM is dynamic, directs morphogenesis, and generates forces to determine tissue shape autonomously, reviewed in ref [71]. However, the mechanical role of ECM, if any, on fate determination in early embryogenesis remains heavily understudied. The initiation of the mechanotransduction pathway is not restricted to stiffness and ECM pathway; it can be mediated via a change in cell surface that leads to activation of mechanosensitive ion channels, like Piezo1 eliciting transcriptional change [16, 72]. Mechanicasitically, tensile force/deformation of the membrane physically activated Peizo1 [16]. Linking Peizo1 or other ion channels to a specific transduction pathway is challenging as ions (e.g., Ca^2+^) are involved in various pathways and cellular responses. For example, the activation of Piezo1 has been linked to the activation of Yap independently of known Hippo upstream activators (Fig. 3b, c) [23, 43]. Nevertheless, proteins that are activated mechanically and independent of biochemical signaling provide a major advantage of being a readout of a given physical force (Fig. 3b).

In addition to the ECM and cell surface, the cytoskeleton is a major component of the mechanotransduction pathway. Certainly, trophectoderm specification is mechanically controlled [73]. Mechanistically, intermediate filaments rich with keratin stabilize F-actin within the apical domain of murine embryos; subsequently, this contributes to the asymmetrical structural component that determines the fate of the trophectoderm [73]. Moreover, actomyosin-dependent contraction in avian skin mediates dermal progenitors, initiating a mechanotransduction pathway in adjacent cells, leading to the fate determination of feather follicles [74]. Hair follicles in murine embryos exhibit actomyosin-dependent rearrangement, where anterior epidermal follicle progenitors relocate to the periphery and posterior cells to the center; this mechanically dependent rearrangement elicits asymmetrical morphology of follicles and predicts cell fate [75]. This actomyosin-dependent rearrangement depends on morphogenesis as an active physical force deriving the rotational cell flow that controls the rearrangement of epidermal follicle progenitors, initiating migration, and patterned fate determination of hair follicles [75]. An additional example of morphogenesis as an active mechanical drive for migration and fate determination can be seen in the case of neural crest cells. Neural crest cells are one of the cell types with the ability to sense the environment and act accordingly; for example, evidence suggests that neural crest migration [76] and differentiation are mediated by mechanical cues [72]. Mechanistically, neural crest cells are able to sense the increase in cell density of the head mesoderm, leading to a rise in mesoderm stiffness from 50 Pa to 150 Pa, which is sensed by neural crest cells via focal adhesions, triggering its migration [52]. Additionally, culturing neural crest stem cells (NCSCs) derived from induced pluripotent stem cells (iPSCs) on different hydrogel stiffness will control the fate outcome of these cells between smooth muscle or glial cells [77, 78]. Lastly, inhibition of Rho-associated kinases (ROCK) and myosin ll resulted in the expansion of neural crest markers (*foxd3* and *Sox8*) during induction in *Xenopus* embryos [79]. The outcome of this study suggests the possible role of mechanical input during neural crest induction and the potential translation of mechanical input via ROCK and myosin ll. Together, these findings shed light on the pivotal role of mechanical input on cell fate and other cellular responses mediated via a cross-talk between a physical force and biochemical signals.

### The nucleus as a mechanosensor

More recently, an additional pivotal role of the cytoskeleton has emerged in the field of biomechanics. It is hypothesized that the cytoskeleton not only acts as a translational component of the mechanotransduction pathway but also has a selective mechano-protection role of the nucleus and chromatin [80, 81]. An example of this can be seen in the depletion of cytoskeleton components (actomyosin, intermediate filaments, Keratin-rich intermediate filaments, Vimentin intermediate filaments, desmin intermediate filaments, and microtubules) that promote nuclear volume expansion, chromatin decondensation, nuclear deformation, nuclear rapture, DNA damage, chromatin organization, and/or high heterochromatin [80–85]. This possible mechano-protective role requires further investigation into whether there is a feedback loop between the cytoskeleton and nucleus or if it occurs autonomously. The possibility of the nucleus regulating the mechanosensitivity of the cells towards a physical force suggests that the nucleus could be an independent mechanosensor that regulates the mechanotransduction pathway. In recent years, the idea that the nucleus is a mechanosensor has gained vast interest and has been the focal point of researchers. Specifically, the LINC complex directly links the cytoskeleton to the nuclear envelope and has shown its role in transmitting force from ECM to the nucleus, leading to gene regulation [86–88]. Mechanistically, mechanosensitive components of the nucleus, nuclear lamins, derive several cellular outcomes in response to changes in stiffness by altering their subcellular localization, post-translational modifications, or their 3D confirmation (Fig. 3d) [89, 90]. Buxboim and colleagues examined the mechanotransduction pathway of the nucleus. They showed that an increase in stiffness leads to a change in the activity of myosin II and, subsequently, de-phosphorylation of lamin A (a nucleoskeletal protein) [90]. More direct evidence that the nucleus can independently regulate force is when its shape is altered [91]. Researchers deformed the nucleus shape by applying pressure with the cantilever of an Atomic Force Microscope and found that these nuclear deformations can regulate the transcriptional activity via Yap independently of upstream regulators such as focal adhesion [91]. A possible mechanism of how the shape of the nucleus regulates mechanical signals is by controlling nucleocytoplasmic transport (NCT) [92]. Nuclear deformation correlates with nucleocytoplasmic transport activity, which regulates protein translocation activity such as Yap. NCT activity was perturbed by osmotic shock or contractility inhibition, reducing NCT activity [92]. This strengthens the idea of the nucleo-dependant mechanotransduction pathway. As the idea of nuclear mechanosensitivity is recent, further investigation is necessary to test similar mechanisms *in vivo* and link this process to cell fate determination during development. Further interplay of physical force and nucleoskeletal is reviewed in reference [19]. Collectively, these studies show the possibility of various physical forces transmitted and regulating cellular response at different convergent points.

## Concluding remarks

This review encapsulates the recent findings on the role of biomechanics on early embryonic fate determination. Researchers have demonstrated the interplay of mechanical cues on cell fate, tissue formation, and function, raising questions about the potential sources of mechanical stimuli during development and how this applied strain guides embryogenesis. More specifically, possible active inputs that regulate embryonic induction of neural plate, neural crest, placodes, or other embryonic cells. Early embryonic development in humans and other species is a highly intricate, organized, multistep, and spatiotemporally controlled process. It constitutes tissue (re-)arrangments, growth, elongation, and more, which could be a source of physical force regulating cell fate. Addressing the question of how cell fate is controlled in early development could lead us to develop new methods for mechanical manipulation, implement new insights into organoids, and gain a new perspective on embryogenesis in health and disease.

## Data Availability

Not applicable.
